# Chemical, Biological, and Ecological Evidence for Aerobic Deoxynivalenol Detoxification in Agronomic Soil-Derived Bacterial Communities

**DOI:** 10.3390/toxins18060273

**Published:** 2026-06-22

**Authors:** Natalia Martínez-Reyes, Rosa E. Cardoza, Estela Melcón-Fernández, Rafael Balaña-Fouce, Lea Brückner, Rocío Montes-Ruiz, Benedikt Cramer, Hans-Ulrich Humpf, Pedro A. Casquero, Santiago Gutiérrez

**Affiliations:** 1 Grupo Universitario de Investigación en Ingeniería y Agricultura Sostenible (GUIIAS), Área de Microbiología, Universidad de León, 24400 Ponferrada, Spain; nmarr@unileon.es (N.M.-R.); re.cardoza@unileon.es (R.E.C.); 2 Departamento de Ciencias Biomédicas, Facultad de Veterinaria, Universidad de León, 24071 León, Spain; emelf@unileon.es (E.M.-F.); rbalf@unileon.es (R.B.-F.); 3 Institute of Food Chemistry, University of Münster, 48149 Münster, Germany; lbrueckn@uni-muenster.de (L.B.); cramerb@uni-muenster.de (B.C.); humpf@uni-muenster.de (H.-U.H.); 4 Grupo Universitario de Investigación en Ingeniería y Agricultura Sostenible (GUIIAS), Instituto de Medio Ambiente, Recursos Naturales y Biodiversidad, Universidad de León, Avenida Portugal 41, 24071 León, Spain; rmonr@unileon.es (R.M.-R.); pacasl@unileon.es (P.A.C.)

**Keywords:** deoxynivalenol, mycotoxins, biotransformation, soil bacteria, mass spectrometry, detoxification

## Abstract

Deoxynivalenol (DON) is a prevalent trichothecene mycotoxin in cereals that poses food and feed safety risks while causing important economic losses. Microbial biotransformation offers a selective, mild strategy for DON detoxification. Here, we screened aerobic soil-derived bacterial communities from diverse agricultural environments, using DON as the sole carbon source for this mycotoxin depletion. More than half of the tested enrichment samples showed a reduced DON signal, as observed by HPLC-UV. To assess the biological relevance, culture extracts were tested for cytotoxicity in HepG2 cells. Z13, a soil sample that depleted DON but produced no other detectable metabolites, showed reduced cytotoxicity, comparable to the negative control. In contrast, samples that depleted DON but produced 3-keto-DON remained toxic. High-resolution LC-MS analysis indicated the formation of metabolites putatively identified as 3-keto-DON in enrichment cultures and 3-epi-DON in a *Devosia* strain culture. Community composition was profiled with 16S rRNA gene amplicon sequencing, which showed that Z13 presented a remarkable drop in diversity upon microbial cultivation, and included genera such as *Devosia*, *Nocardioides*, and *Pseudomonas*. Together, these results provide integrated chemical, biological, and ecological evidence for aerobic DON biotransformation in soil-derived communities, identify pathway products, and highlight practical constraints related to community dependence and storage sensitivity.

## 1. Introduction

Deoxynivalenol (DON) is a mycotoxin produced by *Fusarium* species that frequently contaminates wheat, maize, and barley, leading to recurrent food and feed safety concerns and substantial economic losses [1,2]. Exposure to DON has been associated with reduced feed intake, vomiting, impaired growth performance, intestinal dysfunction, and immune dysregulation in animals [3]. DON binds eukaryotic ribosomes, inhibits protein synthesis, and activates the ribotoxic stress response, driving downstream inflammation and cytotoxicity in exposed cells and tissues [3].

Assessment and monitoring of mycotoxin contamination in cereals and related food products indicate that current levels of DON exposure may pose a health risk, particularly for populations with high cereal consumption. Special attention is needed to ensure food safety, especially for children [2,4]. Moreover, DON contamination in animal feed is widespread and leads to very significant financial losses in the pig production industry [5]. Among animals, pigs are the most sensitive to DON; poultry and ruminants have similar lower levels of sensitivity [3]. Given the prevalence of this mycotoxin and its biological strength, practical detoxification strategies that preserve nutritional quality are urgently needed across grain value chains.

To reduce DON presence in cereals, integrated farm-to-fork measures are implemented, including the use of resistant varieties and optimized agronomic strategies such as crop rotation, correct fungicide use, and moisture control [6]. However, these measures are often not sufficient to effectively prevent DON contamination. As an alternative, detoxification strategies have been explored. Several attempted physical and chemical decontamination methods include heat treatment [7], radiation with gamma rays, ultraviolet-C, and ozone treatment [8,9], but these methods often result in loss of food quality and incomplete detoxification.

In the field, plants usually detoxify DON by glucose conjugation, with DON-3-glucoside (DON-3G) appearing as the main metabolite [10]. Fungi like *Fusarium graminearum* can also transform DON into less toxic forms, mainly by acetylation at carbon 3 (C3) producing 3-acetyl-DON (3-ADON) as a self-defense mechanism [11]. Unfortunately, DON-3G conjugates can be hydrolyzed back to DON in the intestines of animals [12,13], and 3- and 15-ADON are mycotoxins that remain a threat to animal and human health [3].

In contrast to other organisms, it has been described that bacteria have an extensive arsenal to fight DON, and a wide variety of DON-derived metabolites have been identified in bacterial cultures [14,15,16,17]. Currently, bacterial metabolization of DON is considered the most promising detoxification approach due to its specificity and compatibility with mild processing conditions in contrast to chemical and physical detoxification. The most interesting pathways include de-epoxydation, which produces DOM-1, with markedly reduced toxicity; and oxidation–epimerization, with 3-epi-DON as the resulting metabolite, which is also substantially less toxic. Both represent possible detoxification endpoints [18].

DOM-1 predominates in anaerobic microbiomes such as animal intestines, with isolates including *Slackia* sp. D-G6, *Eggerthella* sp. DII-9, and *Eubacterium* sp. BBSH 797 [19,20,21]; nevertheless, one strain has been found in soil that de-epoxydates DON in both aerobic and anaerobic conditions [22]. Notably, the commercial feed additive for mycotoxin reduction, Mycofix plus BBSH^®^ (Biomin, Getzersdorf, Austria), contains the *Eubacterium* sp. BBSH 797 strain as a DON-detoxifying agent. Little information is known about the genes or enzymes involved in trichothecene de-epoxidation.

In contrast, environmental microbiomes, particularly under aerobic conditions, harbor the two-step epimerization of DON: oxidation at C3 to 3-keto-DON followed by reduction to 3-epi-DON [23]. In *Devosia mutans* 17-2-E-8, the responsible enzymes, DepA (a pyrroloquinoline quinone (PQQ)-dependent dehydrogenase) and DepB (an NADPH-dependent dehydrogenase), perform these sequential transformations to yield 3-epi-DON. Across *Devosia* and other environmental isolates, strains can complete one or both steps. Access to cofactors such as PQQ can be limited in pure culture since many *Devosia* strains lack PQQ synthesis ability [24], but their availability can be restored within the microbial consortia [25].

Despite the growing number of bacterial isolates reported to transform DON, limited attention has been given to understanding the complexity of aerobic soil-derived microbial communities that mediate DON biotransformation and detoxification. Existing studies often focus on toxin depletion without adequately assessing the toxicity of transformation products [16,25,26,27], resulting in uncertainty regarding the safety of microbial detoxification pathways. Furthermore, practical and ecological factors influencing community stability and long-term applicability remain poorly understood. Addressing these gaps requires an integrated assessment combining chemical, toxicological, and microbial community analyses.

Most biological DON detoxification reactions target the hydroxyl group at C3, yet common C3 and C15 acetylations and C3 glycosylation are reversible and can regenerate DON under gastrointestinal conditions. Conversely, other modifications like de-epoxidation are more stable and yield less toxic molecules. *Fusarium* trichothecenes are characterized by the presence of a reactive hydroxyl group in C3, but other trichothecene mycotoxins like those produced by *Trichoderma* lack this functional group [28]. This structural diversity raises the possibility that exposure to different trichothecene chemotypes in soil environments may influence the repertoire of microbial detoxification pathways, although direct evidence for such ecological selection remains limited. This possibility is consistent with the principle that selective pressure from toxic compounds in the environment drives the recruitment of specialized microbial pathways, where the specific “complement of enzymes” available for evolution is directly influenced by the structural characteristics of the chemicals present in that niche [29]. We hypothesized that soil samples that contain *Trichoderma* trichothecenes such as harzianum A (HA) could favor bacterial detoxification strategies that do not target C3, e.g., de-epoxidation. Based on this rationale, soil samples with a known presence of trichothecene-producing *Trichoderma* strains, as well as a set of soils treated with HA and an HA-producing strain, were included among the soil samples screened for DON depletion.

In this study, we aimed to: (i) assess the capacity of diverse aerobic agricultural soil-derived bacterial communities to deplete DON; (ii) identify and characterize the resulting transformation products using liquid chromatography–high-resolution mass spectrometry (LC-HRMS), which revealed the putative formation of 3-keto-DON in enrichment cultures and 3-epi-DON in a *Devosia* strain culture, discovering a novel DON degrading strain; (iii) determine the cytotoxicity of the bacterial culture extracts in HepG2 cells; (iv) profile the bacterial community structure through 16S rRNA gene amplicon sequencing; and (v) pursue community simplification strategies and isolation and identification of bacterial consortium members.

## 2. Results

### 2.1. Screening of Soil Samples for DON-Degrading Activity

A multi-methodological approach was used to identify aerobic soil bacteria capable of detoxifying DON. Soil samples were obtained from various sources: (i) fields known to harbor trichothecene-producing *Trichoderma*, (ii) fields with crops infected by *Fusarium*, (iii) soils artificially inoculated with a trichothecene-producing *Trichoderma* strain (T19) and amended with harzianum A (HA), and (iv) soils from diverse cultivars, different from the previous ones (Supplementary Table S1). The screening process included cultivation in a mineral medium supplemented with DON as the sole carbon source (MM-DON) for 7 days. Part of the culture was used for extraction of DON and part of it was cryopreserved with glycerol at −80 °C. Subsequent high-performance liquid chromatography with ultraviolet detection (HPLC-UV) analysis allowed the measurement of DON content.

**Figure 1:**
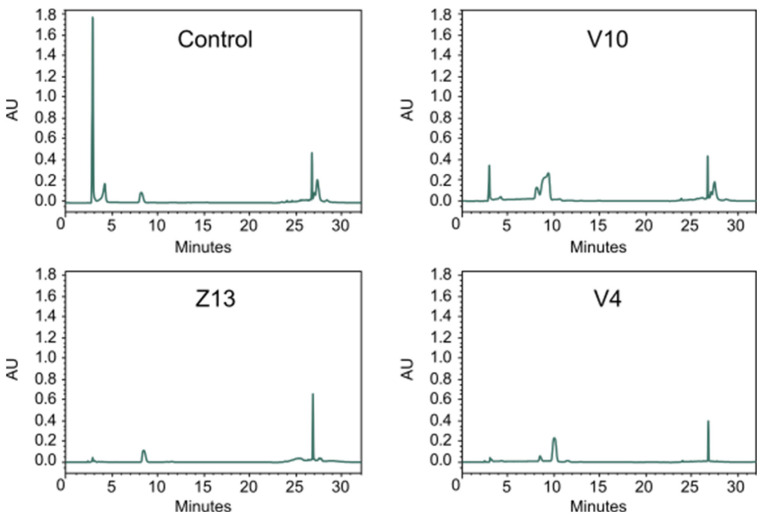
HPLC–UV chromatograms of soil sample screening for DON degradation, showing the four different patterns observed in this analysis. Absorbance at 218 nm in arbitrary units (AUs) is shown. The control (MM + DON) with a prominent DON peak at 3 min RT. V10 culture showing a substantial decrease in the DON peak with a broad new signal at ~9 min RT. Z13 culture showing near-complete loss of the DON peak with no evident new peaks. V4 culture showing near-complete loss of the DON peak with a new different peak at 10 min RT.

In assessing the reduction in DON levels, 27 of the 43 soil samples analyzed showed a > 60% decrease in the DON peak, observed at around 3 min of retention time (RT) (Figure 1, Control). Among these, 12 samples exhibited > 95% elimination of the DON peak. Three distinct chromatographic profiles were identified across the samples after DON transformation. In most cases, a new broad peak appeared at 9–10 min RT, exhibiting an area proportional to the decrease in the DON peak (Figure 1, V10). Three samples, C4, V15, and Z13, displayed DON degradation with no new peaks compared to the control group (Figure 1, Z13), while in one sample, V4, a more defined peak was observed at 10 min RT (Figure 1, V4).

A significant reduction in the DON peak was found in 62% of the analyzed soil samples (Supplementary Table S1). Interestingly, the six soil samples artificially inoculated with *Trichoderma* T19 and amended with HA showed high rates of DON transformation. Despite this, no clear correlation was found between DON degradation capacity and crop type, geographic area, collection date, or previous diseases detected in the crop field. One parameter that determined the capacity of the soil samples to degrade DON was the storage time after the collection date. It was observed that the same samples that showed high rates of DON degradation in the first screening did not reproduce the same results when the analysis was repeated some months later using soil as an inoculum. This could be explained by the loss of natural conditions, which could reduce the viability of part of the soil microbiome, since the soil samples had been stored dry in a plastic bag at 4 °C.

**Figure 2:**
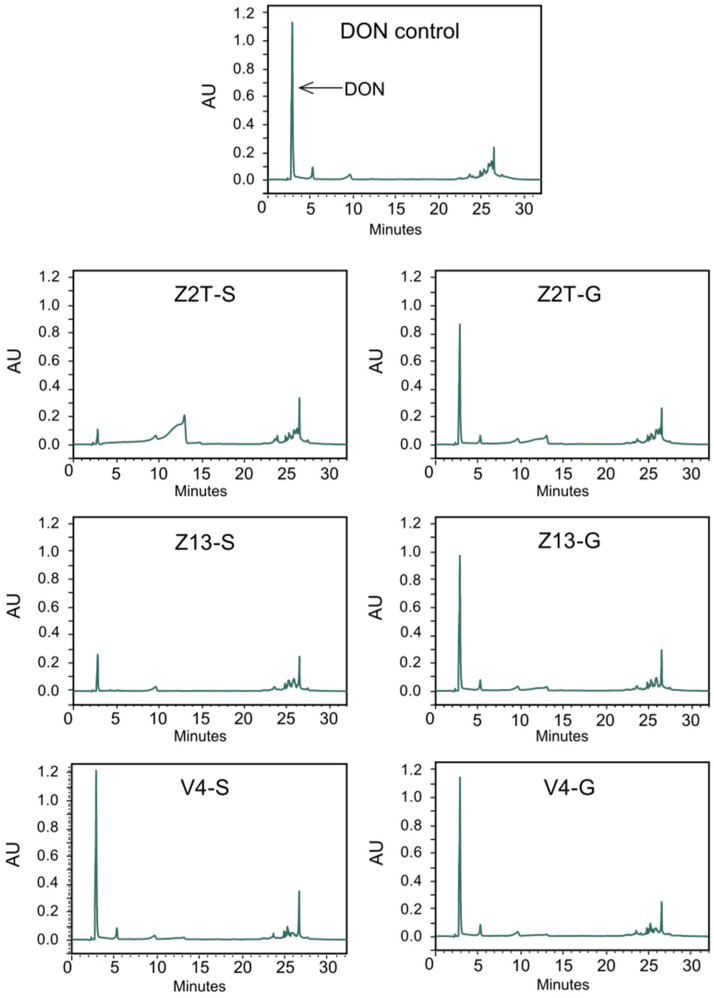
HPLC–UV chromatograms (absorbance at 218 nm in arbitrary units (AUs)) comparing DON in cryopreserved-sample-derived cultures versus soil-derived cultures. The top panel corresponds to the DON control (MM + DON). Each pair of panels corresponds to the same soil sample, with the culture being inoculated from soil (suffix “-S”), or from the cryopreserved sample (suffix “-G”): Z2T19-S (left) and Z2T19-G (right), Z13-S and Z13-G, and V4-S and V4-G.

To further characterize the DON degradation by these soil bacterial populations, the cultures were repeated with the samples that were cryopreserved in glycerol, using the same method as above for cultivation and screening. The comparison between cultures inoculated with soil (-S) and those from glycerol-cryopreserved cultures (-G) evidenced different results (Figure 2), probably related to changes in the bacterial population that could grow better after thawing and subsequent culture rounds. Thus, cryopreservation of bacterial populations came up as a bottleneck of the process.

### 2.2. Toxicity Assessment of DON-Degraded Samples in Cell Culture

To evaluate if these DON degradation results could, in fact, make DON-contaminated material safer, toxicological analyses were performed in HepG2 cells to assess the cytotoxicity of metabolites resulting from the bacterial degradation of DON.

Five of the culture samples inoculated with soil and derived from the cryopreserved cultures described above (see Figure 2) were used for toxicological analysis, each offering valuable insights into the interplay between DON exposure, bacterial presence, and bacterial metabolites. Additionally, controls of the MM without DON (negative control) and MM with DON (positive control) were included. These culture samples were extracted, resuspended in water, sterilized, and added to the cell cultures at 1/2 and 1/4 dilutions. Each extract was tested in biological duplicates.

**Figure 3:**
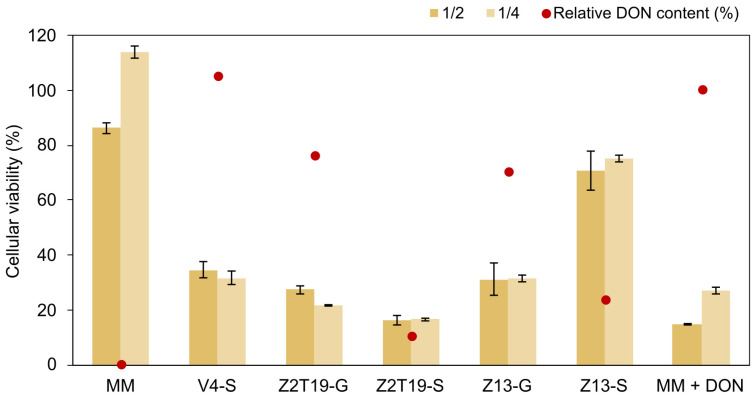
Viability of HepG2 cells after exposure to culture extracts and controls. Cells were treated with extracts from V4-S, Z2T19-G, Z2T19-S, Z13-G, and Z13-S at 1/2 or 1/4 dilution, alongside bacterial culture medium (MM) and medium amended with DON (MM + DON) controls. Viability was normalized to medium negative control (MM). Bars: mean ± SD of two biological replicates. DON content relative to the MM + DON control is also shown.

The HepG2 cell density, i.e., cell survival, was drastically reduced in MM + DON compared to the MM alone, showing that the medium alone did not cause toxicity and confirming the detrimental impact of the mycotoxin on cellular health (Figure 3). Cell growth was significantly impaired in samples V4-S, Z2T19-G, and Z13-G, which correlated with high DON concentrations in the samples. In the case of Z13-S, which significantly degraded DON according to the HPLC-UV results, this bacterial culture did not significantly affect the cellular viability as compared to the MM negative control (t-student of ½ dilution *p* = 0.095), while it significantly improved the viability as compared to the MM-DON positive control (t-student of ½ dilution *p* = 0.008). Interestingly, cells in Z2T19-S showed reduced viability despite presenting lower levels of DON than Z13-S, suggesting that the broad peak appearing at 12 min retention time after DON degradation corresponded to a toxic substance.

### 2.3. Metabolite Identification by LC-HRMS

To identify the metabolites resulting from DON degradation in the studies described above, some cultures were repeated in biological triplicates and analyzed by LC-HRMS, using an ultra-high performance liquid chromatography system coupled to an electrospray ionization source–time-of-flight mass spectrometer (ESI-Q-TOF). For these culture experiments, only the cryopreserved culture samples obtained after the soil screening (suffix “-G” in the previous experiments) were used as an inoculum.

**Figure 4:**
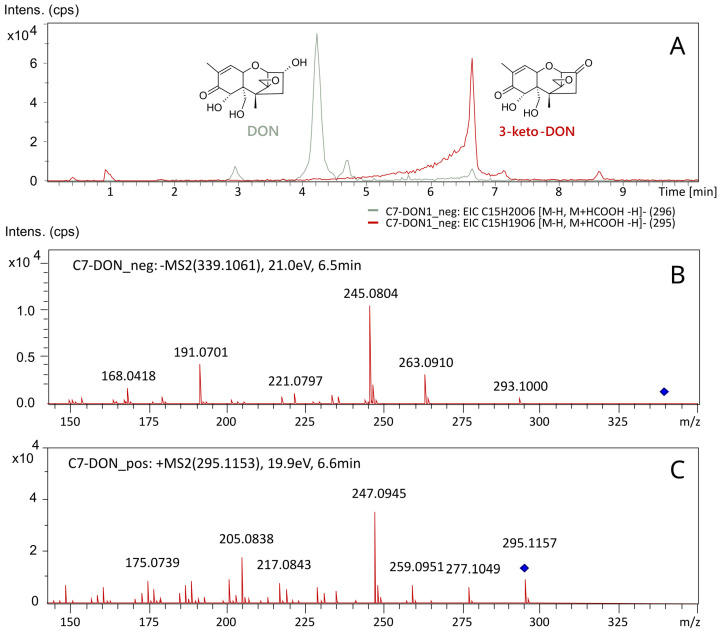
LC-HRMS evidence for DON biotransformation to 3-keto-DON. (**A**) Extracted-ion chromatograms (EICs) for DON (C15H20O6 [M-H, M+HCOOH-H]) in soft green and the putative metabolite 3-keto-DON (C15H19O6 [M-H, M+HCOOH-H]) in red in culture C7. Molecular structure of the indicated compounds is also shown. (**B**,**C**) Product-ion spectra (MS/MS) of the putative 3-keto-DON precursor in negative and positive ion modes, respectively, with diagnostic fragments supporting the 3-keto-DON assignment. For negative ionization mode, the formate adduct is the precursor.

The mass spectrometry results confirmed the conversion of DON. Notably, in sample Z13, DON was below the detection limit, suggesting complete degradation. In samples C7 and V15, the presence of a metabolite matching the exact mass of 3-keto-DON was detected, with its abundance inversely correlating with that of DON. This observation pointed to 3-keto-DON as the likely metabolite resulting from DON degradation in these samples. Other cultures prepared in a further attempt were analyzed, and the same ion appeared after DON degradation (V4, V15, V16, V19 and Z1T19, data not shown). This putative 3-keto-DON metabolite corresponds to the predominant broad peak eluting at 9–10 min when samples were analyzed using the HPLC-UV method. In this method, optimized for metabolite identification, the retention time was 6.6 min (Figure 4A). The MS/MS spectra in negative and positive ionization mode of the parent ion, which corresponded in mass to 3-keto-DON, are presented in Figure 4B,C, respectively. These fragmentation patterns are those expected for 3-keto-DON, as previously reported [26,27]. Although an authentic reference standard was not available, the agreement in accurate mass and fragmentation behavior with published data supports the putative identification of this metabolite as 3-keto-DON.

In sample Z13, the exact-mass ion corresponding to 3-keto-DON was not detected. Nine additional DON-related metabolites (listed in Supplementary Table S2) were screened in Z13 by exact mass, with some tentative matches: DOM-1 as a formate adduct (*m*/*z* 326.1367) at RT 3.54 min; 9,10-dihydro-DON at RT 5.33 and 7.44 min; a predicted hydrolyzed epoxide derivative of DON as a formate adduct (*m*/*z* 314.1366) at RT 0.87 and 2.84 min; and an acetyl-DON isomer at RT 5.62 min. In sample C7, none of these metabolites were detected; however, ions consistent with acetyl-DON were observed at RT 6.65 and 7.91 min. According to prior reports, only two monoacetylated DON species are described: 3-ADON and 15-ADON. While these findings indicate that Z13 may contain minor DON metabolization products, the results are inconclusive, since tandem mass spectrometry data were either unavailable or of insufficient quality to allow putative identification.

To aid in the identification of these metabolites, standards of DOM-1, 3-ADON and 15-ADON were analyzed using the same LC-HRMS method. The standards yielded RTs of 5.5, 7.2, and 7.2 min, respectively, along with their characteristic fragmentation patterns. This confirmed that the ions in Z13 did not correspond to those compounds.

### 2.4. Isolation and Identification of Bacterial Strains

To determine whether DON transformation could be reproduced in simplified microbial systems, bacterial isolates were obtained after cultivation of cryopreserved samples from those soils showing DON transformation without the emergence of new peaks in screening (Z13, C4, V15) or exhibiting tentative DON-derived peaks (V4 with an unidentified peak; Z1T19 with putative 3-keto-DON). As a result, a 16S rDNA (~1500 bp) amplicon was sequenced from 44 isolates (Z13: 14 isolates; V15: 7; V4: 8; Z1T19: 8; C4: 7), detecting genera previously associated with DON degradation, including *Achromobacter spanius* [30], *Stenotrophomonas* [31,32], *Pseudomonas* species [25,33], *Sphingopyxis* sp. [34], and *Pseudoxanthomonas* [34] (see Supplementary Table S3 for details).

Despite their taxonomic affiliation, none of the isolates consistently transformed DON when tested under the same conditions as the cryopreserved cultures. To assess whether DON transformation depended on interactions among community members, synthetic consortia were assembled using both literature-guided and random combinations of isolates, containing up to fourteen strains. However, none of these consortia reproduced the activity observed in the Z13 cryopreserved sample.

An additional attempt to simplify the active community involved pre-inoculum treatment of Z13 with ten antibiotics (Supplementary Table S4), a strategy effective in prior reports [22,32,35]. These treatments also failed to yield cultures capable of DON degradation. In contrast, cultures inoculated with the original Z13 cryostock consistently degraded DON without detectable DON-derived metabolites. Furthermore, replicate derivative cultures from Z13 did not recapitulate this activity, indicating limited reproducibility after community manipulation.

### 2.5. Metataxonomic Analysis of Microbial Communities

To gain a comprehensive understanding of the microbial communities present in the samples and to identify potential contributors to the DON degradation activity that was not observed in the individual isolates, metataxonomic analyses were performed on selected cultures that had previously shown promising results. These included two original soil samples (named -S), Z13 and C4, as well as five glycerol cryopreserved cultures (named -G) derived from Z13, C4, V15, V4, and C7. The analysis of both soil and cryopreserved samples in Z13 and C4 allowed the evaluation of the bacterial community change in the original soil after 7 days of cultivation in MM + DON.

**Figure 5:**
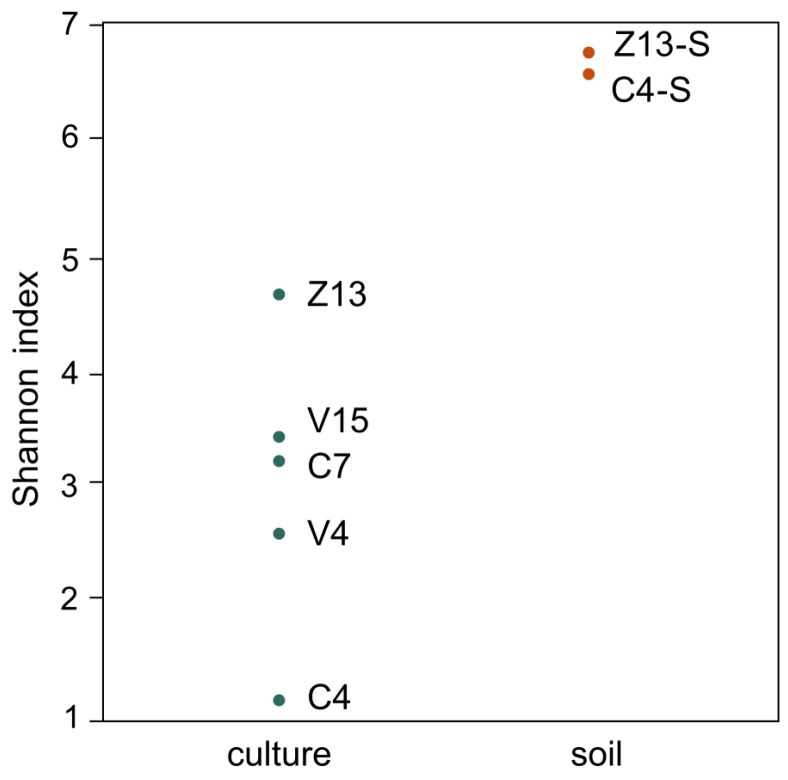
Shannon alpha diversity index values from culture-derived communities (cryopreserved samples) versus soil samples. Points indicate individual samples.

The alpha bacterial diversity of the samples obtained from cultivation and soil was assessed using the Shannon index (Figure 5), which accounts for both the richness and evenness of the taxa present. The analysis revealed clear differences between the two sample types, with soil samples exhibiting higher alpha diversity compared to culture samples. The non-parametric Kruskal–Wallis test indicated a statistically significant difference in alpha diversity between the culture and soil groups (*p* < 0.05), supporting the hypothesis that sample type influences microbial community structure. The individualized graphical representation of each sample allowed for observation of intra-group variability, revealing the remarkable diversity of Z13 in both culture and soil samples.

**Figure 6:**
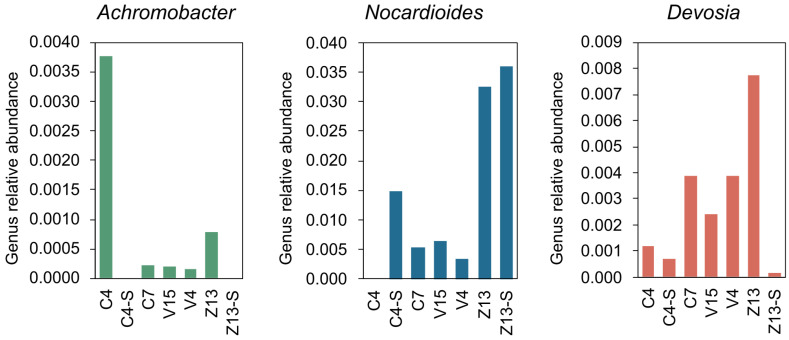
Relative abundances of the genera *Achromobacter*, *Nocardioides*, and *Devosia* across samples.

The relative abundances of the genera *Achromobacter*, *Nocardioides*, and *Devosia* were quantified across all samples (Figure 6) to investigate their potential ecological role and dynamics in relation to DON degradation, since bacteria from these genera have proven to be capable of this detoxification process [26,30,36,37,38,39,40].

*Achromobacter* exhibited a pronounced dominance in the C4 culture sample. In the soil samples, the relative abundance of this genus was negligible. This suggests a successful competitive advantage in the culture microenvironment. Except for Z13, which showed moderate relative abundance levels, all other culture samples displayed only minor levels of *Achromobacter*. *Nocardioides* relative abundance was moderate in certain samples (e.g., C4-S, C7, V15) but reached substantial levels in Z13 in both soil and culture samples, comprising over 3% of the total community in those samples. For *Devosia*, the distribution was more balanced across samples, but with clear peaks in C7, V4, and a striking maximum in Z13.

**Figure 7:**
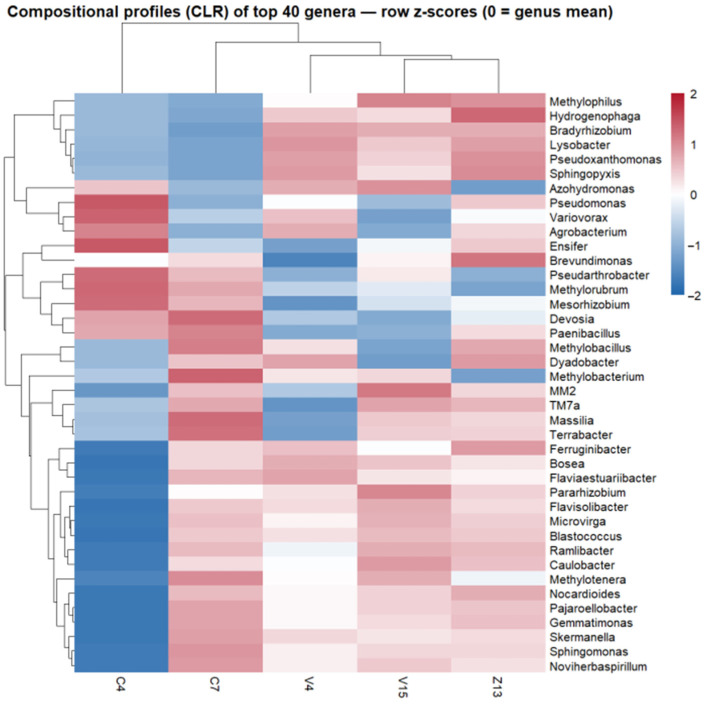
Heatmap displaying compositional profiles of the top 40 bacterial genera across culture samples. Row-wise z-scores of centered-log-ratio (CLR) transformed genus counts are represented; blue indicates below-genus-mean CLR and red indicates above-genus-mean CLR, with hierarchical clustering applied to rows and columns.

To evaluate the compositional differences in microbial communities across the five culture samples, the 40 most variable genera were visualized using a CLR-transformed heatmap (Figure 7) [41]. This scaling highlights variation in genus composition relative to each genus average, providing a complementary viewpoint to the relative abundances illustrated in Figure 6.

Certain genera such as *Methylophilus*, *Hydrogenophaga*, *Bradyrhizobium*, *Lysobacter*, *Pseudoxanthomonas* and *Sphingopyxis* had systematically higher relative abundances (z-score) in samples V4, V15 and Z13 compared to C4 and C7, while genera *Pseudarthrobacter*, *Methylorubrum*, *Mesorhizobium*, *Devosia* and *Paenibacillus* clustered together in a different pattern. These patterns correlate with the relative closeness of the locations where C4 and C7 were collected and the type of cultivar (vineyard), which makes them different from the rest of the samples. Conversely, *Nocardioides* and other genera formed a cluster with consistently lower z-scores in C4 compared to the rest of the samples. In this representation, no clear genus-level compositional patterns emerged among the samples sharing similar chemical outputs. Specifically, C4, V15, and Z13 all completely degraded DON without generating detectable new metabolites during the initial screening, while V4 transformed DON but produced a new unidentified peak and C7 converted DON into the common broad peak putatively identified as 3-keto-DON.

### 2.6. DON Degradation Capacity of a Devosia sp. Strain

As shown by the metataxonomic results, Z13 showed an exceptionally high bacterial diversity, and this sample showed the highest proportion of the *Devosia* genus, which has been shown to degrade DON in different studies. To explore whether this taxonomic signal could be functionally linked to DON metabolism, a *Devosia* strain from a collection (CECT 9973) was used for targeted analysis. This strain was not previously characterized regarding DON degradation and was not isolated from the analyzed consortia. These assays were performed in biological triplicates.

**Figure 8:**
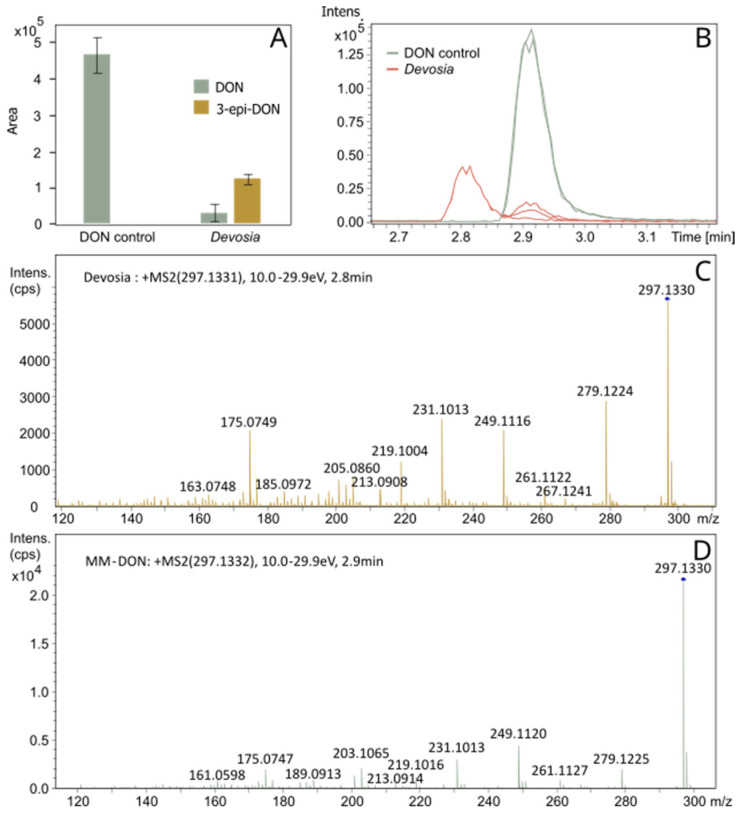
LC-HRMS putative identification of 3-epi-DON produced by *Devosia* sp. CECT 9973. (**A**) Bar chart of integrated peak areas showing DON depletion and formation of a co-isobaric product in *Devosia* cultures relative to the DON control (MM + DON). (**B**) Extracted-ion chromatogram (EIC) overlay: the DON control displays this mycotoxin as a dominant peak at ~2.91 min, whereas the *Devosia* sample exhibits a reduced DON peak and an earlier peak at ~2.81 min consistent with 3-epi-DON. (**C**) MS/MS spectrum acquired at 2.81 min from the *Devosia* sample with precursor [M + H]^+^ at *m*/*z* 297.133 (C15H20O6) and characteristic fragments (e.g., *m*/*z* 279, 261, 249, 231, 175) supporting the epimer assignment. (**D**) MS/MS spectrum from the DON control at 2.91 min showing the same precursor and fragment series for direct comparison with the *Devosia* product.

After 7 days of incubation, the DON content was almost completely degraded, as assessed by LC-HRMS, with the average intensity of the DON peak decreasing from 465 ± 48 kcps in the untreated control to 30 ± 24 kcps in the *Devosia* culture (Figure 8A). This corresponds to a reduction of approximately 93.5%. Alongside the reduction in DON content, a new compound was detected in the *Devosia* samples with an average intensity of 121 ± 13 kcps. This compound had the same mass as DON but exhibited a slightly lower RT of 2.81 min, compared to 2.91 min for DON. In two of the three replicates, both peaks were clearly observed in the extracted-ion chromatogram corresponding to the exact mass of DON (Figure 8B). The new compound (RT 2.81 min) appeared in proportion to the reduction in DON (RT 2.91 min), suggesting a potential conversion between the two. This compound was tentatively identified as 3-epi-DON, a common bacterial metabolite associated with DON degradation [42,43,44]. To verify this, auto MS/MS analysis was performed to obtain the fragmentation pattern of the compound. The resulting MS/MS spectra from three replicates exhibited ion peaks highly similar to those of the DON control (Figure 8C,D). Although an authentic reference standard was not available for definitive confirmation, the agreement between the observed mass, retention behavior, fragmentation pattern, and previously reported DON epimerization products supports the putative identification of this metabolite as 3-epi-DON. In this configuration, the hydroxyl group at the C-3 position adopts the S configuration, placing it on the same face of the molecule as the epoxide ring. This stereochemical arrangement likely increases the compound’s polarity slightly, which is consistent with its earlier elution (by 0.1 min) compared to DON in our LC method. This behavior agrees with previous studies, such as [45], where 3-epi-DON eluted earlier than DON under LC conditions with a gradient of increasing polar solvent.

Regarding the presence of other common DON derivatives, no signals corresponding to 3-keto-DON, acetylated forms of DON, or glycosylated DON were detected in the *Devosia* samples based on their exact masses. In other studies on *Devosia* strains, 3-keto-DON is frequently observed as an intermediate in the conversion of DON into 3-epi-DON [39,46], and as a DON transformation product [38]; however, in this work, 3-keto-DON was not detected at all.

## 3. Discussion

This study successfully obtained aerobic soil samples able to degrade DON using a multi-method approach. HPLC-UV screening established that more than half of the tested soils enabled substantial DON loss over a 7-day culture, with the main chromatographic signature showing a broad peak putatively identified as 3-keto-DON and near-complete DON disappearance without visible new UV peaks in several cultures, indicating multiple transformation fates. Structural elucidation by LC-HRMS allowed for assigning the metabolite which appeared in most samples to 3-keto-DON based on exact mass, adduct forms, and MS/MS fragments in both ionization modes. These outcomes align with known aerobic DON biotransformations, where C3 oxidation to 3-keto-DON and subsequent epimerization to 3-epi-DON are frequently observed [26,33,47]. This finding reflects that many bacteria from soil can degrade DON in their ecological niche. Notably, microbial degradation activity was influenced by storage conditions, with cryopreservation affecting bacterial viability and community composition. This storage sensitivity could cause loss of viability of key degraders, disruption of interspecies interactions needed for multi-step metabolism, or destabilization of labile enzyme systems and cofactors during freeze–thaw cycles, and it sets an important constraint for preserving functional consortia.

The loss of activity after cryopreservation and subsequent cultivation rounds could be caused by bacterial community dynamics being affected by the selective pressure of cryopreservation conditions, rather than by DON degradation. Cryopreservation in glycerol at −80 °C was selected because it is a standard and widely used method for the long-term preservation of both bacterial isolates and mixed microbial communities. However, we acknowledge that this approach may affect the recovery of complex microbial consortia. Previous studies have reported that cryopreservation and subsequent revival can alter community composition and impair the recovery of community-level functions, even when dominant taxa remain viable [48]. Attempts to reproduce DON degradation with isolated bacterial strains or simplified consortia were unsuccessful, underscoring the possible importance of unknown community members or metabolic cooperation. This is a common phenomenon in studies where microbial DON degradation is pursued [31,49,50]. In the experiments with *Devosia* CECT 9973, a slow growth rate was observed in different media (data not shown), and the colonies of this strain were punctiful in solid media and very small compared to most isolated strains found in this study, which grow faster and produce bigger colonies.

Metataxonomics analysis showed an important reduction in bacterial diversity after cultivation according to the alpha diversity results. A previous study from Kenngott et al. [47] shows that soils with large microbial communities and a low fungi-to-bacteria ratio exhibit more efficient DON transformation, highlighting the natural capacity of soil microbes to mitigate this agricultural contaminant. To illustrate patterns at the genus-level community structure, both relative abundance barplots and a CLR (centered-log-ratio) row z-score heatmap were examined. The barplots (Figure 6) showed the absolute genus relative abundance across culture samples, for example, revealing that *Devosia* reached its highest proportional abundance in sample Z13, while C4 and C7 exhibited much lower abundances for this genus. The relative abundance of *Devosia* was quite low in Z13-S, possibly due to the exceptionally high overall diversity of this soil sample, with this meaning that after cultivation, *Devosia* was remarkably enriched in the culture sample Z13. In contrast, the heatmap (Figure 7) emphasized differences in abundance patterns among samples rather than absolute relative abundance. Consequently, genera that were highly abundant in one sample did not necessarily appear as the most prominent features in the heatmap. Despite these differences in representation, both analyses support the conclusion that *Devosia* was enriched during cultivation of the Z13 community. However, enrichment of genera previously associated with DON degradation, including *Devosia*, *Achromobacter*, *Nocardioides*, *Pseudomonas*, *Lysobacter*, *Pseudoxanthomonas* and *Sphingopyxis*, was also observed [33,34,37]. The tight clustering of *Lysobacter*, *Pseudoxanthomonas* and *Sphingopyxis* genera suggests potentially related ecological roles or a joint response to culture conditions. In previous reports where diversity was also assessed, the cultivation rounds, with subsequent diversity reduction, helped in selecting the microorganisms able to perform DON detoxification [34,51,52]. To identify bacterial taxa lost during cultivation, as well as those that predominate without contributing to DON degradation, future experiments should analyze a broader range of samples. These should include soil, intermediate enrichment cultures capable of degrading DON, and cultures in which this ability has been lost.

In the singular case of sample Z13, DON disappearance without detectable metabolites suggests that DON may have been transformed into metabolites that were not detected or may have undergone possible complete mineralization, which has been hypothesized yet not directly demonstrated in previous reports [49]. The extraordinary diversity of this sample, as assessed by the metataxonomic analysis, may have contributed to synergies between bacteria that led to this effect. In some reports [23,37,40], it has been demonstrated or hypothesized that some bacteria produce metabolites that other bacteria can use for their own metabolism. Here, we observe a high correlation between bacterial diversity and DON degradation.

The biological relevance of these transformations is underscored by HepG2 cytotoxicity outcomes, which revealed that not all DON-depleted extracts conferred reduced toxicity; notably, Z13-S extracts preserved cell viability relative to the medium and exceeded the MM + DON control, whereas other extracts decreased viability, implicating either residual DON or concurrent production of cytotoxic bacterial metabolites, including the putatively identified compound 3-keto-DON, in Z2T19-S. Other studies have also reported DON depletion with a toxic outcome [34]. These results emphasize that detoxification claims cannot rely solely on DON depletion and require an additional biological assessment.

The *Devosia* sp. strain (CECT 9973), used as a genus-level functional probe, demonstrated nearly complete DON degradation and the putative formation of 3-epi-DON, evidenced by co-isobaric [M + H]^+^ 297.133, earlier retention, and a characteristic fragment series consistent with the epimer in LC-HRMS analyses, supporting its potential role in detoxification. This finding aligns with previous reports implicating *Devosia* species as candidate DON-degrading bacteria [23]. In these reports, *Devosia* has been able to transform DON into 3-epi-DON and 3-keto-DON. This latter metabolite can be observed as an intermediate in the conversion of DON into 3-epi-DON [39,46], but also appears as a final DON transformation product [38]. In our experiments, in cultures of the pure *Devosia* CECT 9973 strain, pre-grown in rich media for 1 day and exposed to DON in minimal media for 7 days, we observed DON transformation into 3-epi-DON. This metabolite is considered a low-toxicity, safe DON degradation product, as assessed in multiple models, including in vitro assays with cells and in vivo tests with mice and piglets [18,53,54], and interestingly, 3-keto-DON was not detected at all. Notably, 3-epi-DON was not detected in Z13 cultures, suggesting that *Devosia*-associated epimerization is unlikely to be the only mechanism underlying DON depletion in this consortium.

Using native microorganisms as detoxification agents is challenging, since their growth requirements and detoxification activities are tuned to their original ecological niches and often do not translate to industrial settings [49,50]. Defining the underlying metabolic pathways and key enzymes would enable transfer of these functions into robust hosts through metabolic engineering. From an application standpoint, the data support the feasibility of DON mitigation strategies based on aerobic soil consortia (Z13) and selected strains (*Devosia* CECT 9973) but emphasize three prerequisites: robust verification that transformation products are less toxic than DON in relevant biological systems; preservation of functional activity during storage and deployment; and reproducible performance.

This study faced limitations including incomplete metabolite characterization, since only MS/MS data was used for metabolite identification. The absence of authenticated standards for all products and lack of orthogonal confirmation (e.g., NMR) reduce the robustness of these results and suggest future validation steps. Also, there were challenges in isolating all active degraders, variable reproducibility due to constraints related to soil sample heterogeneity, and controlled lab conditions differing from natural soil ecosystems. Further research is needed to overcome these challenges.

## 4. Conclusions

This work provides integrated analytical, biological, and ecological evidence that aerobic soil communities harbor DON biotransformation potential, with evidence of total DON biological detoxification in some cases, demonstrated by the absence of detectable DON-related metabolites. Pathway derivatives such as 3-keto-DON and 3-epi-DON were putatively identified in representative cultures and isolates. Toxicological testing demonstrated that DON depletion alone is insufficient, as 3-keto-DON-producing cultures retained cytotoxicity, whereas Z13 depleted DON without detectable toxic effects. Analysis of community composition identified bacterial genera associated with DON transformation and revealed a marked reduction in microbial diversity during cultivation.

Despite these promising findings, practical application remains constrained by the dependence of degradation activity on microbial community structure and the sensitivity of these communities to storage conditions. Short-term applications may include the identification of active strains or consortia and the screening of enzymes involved in DON transformation under controlled conditions. Major limitations include the instability of functional interactions, incomplete understanding of the underlying enzymatic pathways, and the lack of robust, scalable systems for maintaining activity. Future research should focus on identifying the enzymes and metabolic pathways responsible for DON transformation within complex microbial consortia, elucidating the mechanisms underlying community-dependent activity, and optimizing stable, effective microbial consortia, strains, or enzyme-based systems for practical DON detoxification in agricultural and industrial settings.

## 5. Materials and Methods

### 5.1. Sample Collection from Soil

Sampling was designed to represent diverse crop and disease conditions. Soil samples were collected from diverse agricultural environments representing different contamination and ecological contexts: fields associated with *Fusarium* infection, fields harboring trichothecene-producing *Trichoderma*, soils amended with the trichothecene harzianum A (HA) and artificially inoculated with an HA-producing *Trichoderma arundinaceum* strain (T19), and additional agricultural soils from various cultivars and locations.

For soil sample collection, 5–10 spots were randomly chosen for each sample. The top layer of soil was removed, and soil was collected from up to 20 cm deep. The total amount of soil collected for each sample was approximately 500 g. The soil samples were dried at room temperature and sieved through a 2 mm mesh. The information collected about each sample included sampling date, crops that were cultivated at that moment, previously detected diseases in the crops, and location of the field (Supplementary Table S1). Samples were collected from June 2021 to July 2023. Three of the collected samples, Z1, Z2 and Z6, were supplemented with HA or co-inoculated with the HA producer strain T19. Thus, 12 g of soil was added to 5 mL of sterile Milli-Q^®^ water (Merck KGaA, Darmstadt, Germany) and 1200 μg of HA (e.g., Z1HA) or 10^8^ spores of T19 (e.g., Z1T19). These mixtures were disposed in Petri dishes that were incubated in an incubation chamber under a photoperiod (16/8 light/darkness) at 25 °C, and 5 mL of sterile Milli-Q^®^ water was added once a week for 3 weeks. After this time, the soil was collected for further analysis (Supplementary Table S1).

### 5.2. Bacterial Cultures for DON Degradation Assays

For bacterial growth during DON depletion screening, the mineral media (MM) [50] consisted of the following composition: 0.8 g/L K_2_HPO_4_, 0.2 g/L KH_2_PO_4_, 0.2 g/L MgSO_4_, 1.0 mg/L CaCl_2_, 1.5 g/L NH_4_Cl, 1.0 mg/L FeCl_3_, and 2 mL/L of trace element solution TS2, with the pH adjusted to 7.2. TS2 contained (g/L) ZnSO_4_·7H_2_O, 0.1; MnCl_2_·4H_2_O, 0.03; H_3_BO_3_, 0.3; CoCl_2_·6H_2_O, 0.2; CuCl_2_·2H_2_O, 0.01; NiCl_2_·6H_2_O, 0.02; Na_2_MoO_4_·2H_2_O, 0.9; and Na_2_SeO_3_, 0.02.

For soil screening, 4 mL of MM amended with 50 μg/mL of DON (MM + DON) was inoculated with 0.2 mL of a 1/10 dilution of soil in Milli-Q^®^ water and incubated at 30 °C and 180 rpm in an orbital shaker for 7 days. For every cultivation batch, a reference of 4 mL of MM + DON with 0.2 mL of Milli-Q^®^ water was included (100% DON control sample). At the end of this period, 1 mL of each culture was used for HPLC-UV analysis, with 0.5 mL for culture preservation. The cryopreserved culture was prepared by combining 0.5 mL of culture and 0.5 mL of an 80% glycerol solution and stored at −80 °C until further use.

Cryopreserved cultures were prepared by growing a pre-inoculum of 40 μL in 4 mL of MM + 1% glucose for 24 h in the incubation conditions described above. This pre-inoculum was centrifuged, and the cells were used for inoculation of MM + DON, following the same incubation procedure as that for the soil samples. For the *Devosia* CECT 9973 strain and the bacterial isolates obtained in the study, the pre-inoculum was prepared as follows: a colony of the strain was grown in 4 mL of Yeast Mannitol medium (2 g/L yeast extract, 10 g/L mannitol, 0.1 g/L NaCl, 0.5 g/L K_2_HPO_4_, and 0.2 g/L MgSO_4_·7H_2_O in distilled water) in the case of *Devosia* CECT 9973, and MM + 1% glucose for the other strains, until the medium was visibly turbid.

### 5.3. DON Content Assessment by HPLC-UV

Culture samples were extracted twice in ethyl-acetate 1:1. In total, 1 mL of the culture sample was mixed with 1 mL of ethyl acetate by vortexing for 1 min. After centrifugation, the supernatant was collected, and 1 mL of ethyl acetate was added to the aqueous phase. Centrifugation was repeated and the supernatant was again collected. Finally, the solvent was evaporated from the supernatant in a SpeedVac™ vacuum concentrator (Thermo Fisher Scientific, Waltham, MA, USA), and the solid debris was resuspended in 100 μL of acetonitrile and filtered through a 0.2 μm syringe-driven filter unit (PTFE membrane, hydrophobic, Millex-FG, Merck KGaA, Darmstadt, Germany). The concentrated samples were analyzed in a Waters™ Alliance HPLC-UV device (Waters™, Milford, MA, USA) using a reverse-phase XBridge C18 column (130 Å, 3.5 μm, 4.6 × 150 mm; Waters™) and analyzed using the Empower 2 software, build 2154 (Waters™). The mobile phase consisted of water (A) and acetonitrile (B), with a linear gradient program as follows: 0–18.0 min, 13–17% B; 18.0–23.0 min, 17–80% B; 23.0–28.0 min, 80–13% B; and 28.0–32.0 min, 13% B. The flow rate was set to 0.6 mL/min, with an injection volume of 20 μL, without temperature control. Absorbance at 218 nm was monitored. For calculation of DON degradation, the DON peak area was compared to the peak area of the DON control in each screening batch.

### 5.4. In Vitro Cytotoxicity Testing Against Mammalian Cell Cultures

For cytotoxicity testing, culture broths from bacterial isolates were extracted as follows: 1 mL aliquots of each bacterial culture were collected after incubation and subjected to extraction with ethyl acetate, following the same procedure as that described for HPLC-UV analysis. After solvent evaporation, the resulting residues were resuspended in sterile Milli-Q^®^ water to a final volume of 1 mL, and the extracts were sterilized by filtration through 0.2 μm pore-size membranes.

HepG2 cells (human hepatocellular carcinoma cell line) were seeded in 96-well plates at a density of 10,000 cells per well in 100 μL of 2× DMEM medium (Gibco, Thermo Fisher Scientific, Waltham, MA, USA). After overnight incubation at 37 °C with 5% CO_2_ to allow cell attachment, 100 μL of the prepared bacterial culture extract was added per well. Each extract was tested in duplicate. Experimental controls included a cytotoxic positive control (0.4% H_2_O_2_), a negative control (100 µL sterile water), and a medium control (1× DMEM). Additionally, a reference control of DON was included at final concentrations of 80, 40, and 20 μM per well.

Following addition of the treatments, cells were incubated for 72 h at 37 °C and 5% CO_2_. Subsequently, 20 μL of AlamarBlue reagent (Invitrogen (Carlsbad, CA, USA), Thermo Fisher Scientific) was added to each well according to the manufacturer’s instructions. After 4 h of incubation under the same conditions, fluorescence was measured using a Varioskan Lux microplate reader (Thermo Fisher Scientific) with excitation/emission wavelengths set at 560/590 nm.

The results were analyzed by normalizing the fluorescence readings of each sample relative to the control medium (considered as 100% cell viability). The relative viability (%) of HepG2 cells exposed to each bacterial extract and to the DON standards was calculated accordingly.

### 5.5. Metabolite Identification by LC-HRMS

For sample preparation, 0.1 mL aliquots of each bacterial culture were mixed thoroughly with 0.9 mL of acetonitrile and stored at 4 °C. Prior to analysis, these samples were evaporated to dryness using a SpeedVac™ vacuum concentrator (Thermo Fisher Scientific). For reconstitution, each dried sample was resuspended with 1 mL of the initial mobile phase for the analysis, consisting of water and acetonitrile (95:5, *v*/*v*) supplemented with 0.1% formic acid. The mixture was vortexed for 30 s and sonicated in an ultrasonic bath for 5 min. The reconstituted samples were subsequently centrifuged at 15,000 *g* for 5 min at room temperature. A 400 μL aliquot of the resulting supernatant was transferred into glass vials for LC-HRMS analysis. The remaining supernatant was stored at −20 °C for potential further analyses. Samples were analyzed directly without dilution.

Chromatographic separation was conducted using an Elute UHPLC system (Bruker, Billerica, MA, USA) fitted with a NUCLEODUR Phenyl-Hexyl analytical column (100 × 2 mm, 3 μm) and a corresponding guard column (MACHEREY-NAGEL GmbH & Co. KG, Düren, Germany). The mobile phase comprised acetonitrile with 0.1% formic acid (solvent A1) and water with 0.1% formic acid (solvent B1). Separations were carried out using a linear gradient elution program, as follows: 0.0–3.0 min, 5% A1; 3.0–10.0 min, 5–40% A1; 10.0–15.0 min, 40–95% A1; 15.0–16.0 min, 95–5% A1; and 16.0–20.0 min, 5% A1, at a flow rate of 0.5 mL/min, maintaining the column at 40 °C.

The mass spectrometric detection was performed on a Bruker Impact II Quadrupole Time-Of-Flight (Q-TOF) instrument equipped with an electrospray ionization source operating either in positive or negative ionization mode. Data were acquired in auto MS/MS mode, enabling automatic selection and fragmentation of precursor ions based on their intensity in the survey scan. Source settings were as follows: capillary voltage of 4500 V, end plate offset of 500 V, nebulizer pressure of 2.0 bar, dry gas flow of 10.0 L/min, and dry temperature of 220 °C. Full-scan MS data were collected over an *m*/*z* range of 30–1000 at a 2 Hz spectral rate. Fragmentation in the collision cell was performed at a collision energy of 10 eV, with a quadrupole ion energy of 4.0 eV and a low-mass cutoff of 90 *m*/*z*. Calibration of the mass spectrometer was performed using sodium formate, introduced at 3 μL/min. Data acquisition and processing employed both Bruker DataAnalysis 6.1 and Metaboscape version 2024b software.

### 5.6. Isolation and Molecular Identification of Bacterial Strains

The cryopreserved samples were serially diluted with sterile Milli-Q^®^ water and 10^−4^ and 10^−5^ dilutions were cultivated in TSA (Tryptic soy broth + 1.5% agar (Sigma Aldrich, Merck KGaA, Darmstadt, Germany)) to obtain isolated bacterial colonies. Genomic DNA was extracted from a single colony using the Bacterial & Yeast Genomic DNA Purification Kit (EURX Sp. z o.o., Gdańsk, Poland) according to the manufacturer’s instructions. The purified DNA was stored at −20 °C until further use.

For amplification of the 16S rRNA gene fragment, the following primers were employed: 27F (5′-AGAGTTTGATCMTGGCTCAG-3′) and 1492R (5′-GGTTACCTTGTTACGACTT-3′).

PCR reactions were performed with a ProFlex PCR System thermal cycler (Thermo Fisher Scientific). Each 50 μL reaction contained DreamTaq DNA polymerase kit components (Thermo Fisher Scientific), 20 pmol of each primer, and 1 μL of template DNA. The thermal cycling protocol consisted of an initial denaturation at 95 °C for 30 s, followed by 35 cycles of denaturation at 95 °C for 30 s, annealing at 57 °C for 30 s, and extension at 72 °C for 1 min. A final extension was carried out at 72 °C for 10 min.

Amplified PCR products were analyzed by agarose gel electrophoresis. PCR products were then purified using the NucleoSpin Gel and PCR Clean-up Kit (MACHEREY-NAGEL, Germany), following the manufacturer’s procedure.

Purified PCR amplicons were sequenced using the BigDye Terminator v3.1 Cycle Sequencing Kit (Thermo Fisher Scientific) and an ABI 3500 automated capillary sequencer (Thermo Fisher Scientific), according to the manufacturer’s recommendations. Bacterial identification was performed by submitting the obtained consensus sequences to the GenBank database (NCBI, http://www.ncbi.nlm.nih.gov, accessed on 18 June 2024) using the BLAST algorithm (http://www.ncbi.nlm.nih.gov/BLAST, accessed on 18 June 2024).

### 5.7. Metataxonomic Analysis

DNA metabarcoding analyses were carried out by AllGenetics & Biology SL (www.allgenetics.eu, accessed on 7 September 2025). Genomic DNA was extracted from each sample using the PowerSoil Pro kit (QIAGEN N.V., Venlo, Netherlands), following the manufacturer’s instructions. To monitor potential reagent or laboratory contamination, an extraction blank was included and processed in parallel with the samples. DNA concentrations were determined using the Qubit dsDNA High Sensitivity Assay kit (Thermo Fisher Scientific).

To characterize prokaryotic community composition, a ~465 bp fragment of the 16S rRNA gene (encompassing the V3-V4 regions) was amplified using primers Bakt_341F (5′-CCTACGGGNGGCWGCAG-3′) and Bakt_805R (5′-GACTACHVGGGTATCTAATCC-3′) [55]. The first PCR step was performed using Supreme NZYTaq 2× Green Master Mix (NZYTech, Lda., Lisboa, Portugal) and ultrapure water, and the index sequences for multiplexing were added in a second PCR.

PCR products were purified using Mag-Bind RXNPure Plus magnetic beads (Omega Bio-tek, Inc., Norcross, GA, USA). Libraries were quantified with the Qubit dsDNA HS Assay Kit, pooled in equimolar amounts, and sequenced on an Illumina NovaSeq 6000 system (Illumina Inc., San Diego, CA, USA) using 250 bp paired-end chemistry, targeting a minimum yield of 1 gigabase.

For quality control, raw sequencing data were subjected to adapter trimming using Cutadapt v3.5 [56] and sequencing quality was assessed with FastQC [57]. Primer sequences were further removed with Cutadapt (via QIIME 2, release 2024.10v) [58] before denoising and ASV (amplicon sequence variant) inference using the DADA2 pipeline [59]. DADA2 was used to filter low-quality reads, trim reverse reads at position 240 (based on quality profiles), learn error rates, dereplicate the dataset, infer ASVs, merge paired reads with a minimum 12 bp overlap, and remove chimeric sequences.

Taxonomic assignment of ASVs was performed using a pre-trained classifier based on the SILVA reference database (release July 2024; [60]). Singleton ASVs (represented by only one sequence across the entire dataset) were excluded and ASVs present at a frequency below 0.01% in any given sample were removed. Further, ASVs that could not be resolved beyond the dominion level (“d__Bacteria”) or remained unidentified were excluded. Any ASVs detected exclusively in the negative control were discarded from the final dataset.

### 5.8. Bioinformatic and Statistical Analyses of Microbial Communities

Diversity indexes were calculated from the final ASV count table using the R package vegan [61]. Differences in alpha diversity (Shannon index) between culture and soil samples were assessed using the non-parametric Kruskal–Wallis test (implemented in R).

To calculate the relative abundance of selected genera per sample, ASVs annotated as the target genus (e.g., *Devosia*) were selected from the taxonomy table, and counts were then normalized by dividing each value by the total number of reads in its respective sample, yielding the relative abundance for each ASV per sample. The relative abundances of all ASVs belonging to the genus were then summed for each sample, resulting in the total relative abundance of the genus per sample.

Genus-level compositional analyses were performed using centered-log-ratio (CLR) transformation, which addresses the compositional nature of sequencing data by scaling counts relative to the geometric mean abundance within each sample [41]. The heatmap was constructed using the filtered ASV count tables. The ASVs were labeled with sample metadata and taxonomy, and only culture-derived samples were retained. ASVs lacking an assigned genus were removed. Genus-level count matrices were built by summing ASV counts per sample within each genus, producing a samples-by-genera table. A CLR transform was applied to the genus count matrix after adding a pseudocount of 1, computing per-sample proportions, taking natural logarithms, and centering by the per-sample mean of log-proportions; this yielded vectors with zero sum that mitigated false correlations due to the constant-sum constraint in sequencing data [41].

Genera were ranked by median absolute CLR values across samples, and the top 40 genera were selected. For visualization, each selected genus (row) was scaled to z-scores by subtracting its row mean and dividing by its row standard deviation, with zero-variance rows safeguarded by a unit denominator. The z-scored matrix was visualized with a symmetrical break range centered at 0, and hierarchical clustering enabled both rows and columns to group similar genera and samples using pheatmap [62]. Processing and visualization were performed in R.

### 5.9. DON Content Assessment by LC-HRMS

Samples were processed by LC-HRMS, as described above for metabolite identification. Chromatographic separation was performed using an Elute UHPLC system (Bruker, Billerica, MA, USA) fitted with the same column setup as that for metabolite identification by HPLC-HRMS. The mobile phase comprised water with 0.1% formic acid (solvent A) and acetonitrile with 0.1% formic acid (solvent B). Separations were conducted using a stepwise gradient elution program as follows: 0.0–0.25 min, 95% A and 5% B; 0.25–0.50 min, 80% A and 20% B; 0.50–6.00 min, 0% A and 100% B; 6.00–8.00 min, 0% A and 100% B; 8.00–8.10 min, 95% A and 5% B; 8.10–11.00 min, 95% A and 5% B. The flow rate was maintained at 0.25 mL/min throughout the run and the column oven was set to 40 °C.

Detection was carried out on a Trapped Ion Mobility Spectrometry–Time-Of-Flight system (Bruker), operated in Q-TOF mode without ion mobility separation, using electrospray ionization in positive mode. Data were acquired in bbCID (broadband Collision-Induced Dissociation) mode. Internal mass calibration was achieved using sodium formate. Key MS parameters included capillary voltage of 4500 V, end plate offset of 500 V, nebulizer pressure of 3.0 bar, dry gas flow of 8.0 L/min, dry temperature of 220 °C, mass range of *m*/*z* 50–1000, and spectra rate of 2.0 Hz. Fragmentation was produced at a collision cell energy of 7 eV. Additional tuning parameters included Funnel 1 RF of 250.0 Vpp, Funnel 2 RF of 250.0 Vpp, quadrupole ion energy of 4.0 eV, collision RF of 250.0 Vpp, transfer time of 25.0 μs, and pre-pulse storage time of 8.0 μs. Raw data were processed and analyzed using Bruker DataAnalysis 6.1 and TASQ software 2025 version.

## Data Availability

The original contributions presented in this study are included in the article/Supplementary Materials. Further inquiries can be directed to the corresponding author(s).

## Supplementary Materials

The following supporting information can be downloaded at: https://www.mdpi.com/article/10.3390/toxins18060273/s1, Table S1: Soil sample metadata used for DON biodegradation screening. The table lists each sample code, DON degradation percentage with respect to the batch’s control, crop type (e.g., beans, hop, vine), origin/collection location, *Trichoderma* presence/identity and collection date; entries include field soils and in vitro soil cultures inoculated with *Trichoderma* and harzianum A. N/A indicates that *Trichoderma* presence was not analyzed.; Table S2: DON-related metabolites targeted by LC-HRMS, listing compound name, molecular formula, and calculated monoisotopic exact mass [63]; Table S3: 16S rDNA BLAST/NCBI identification of colony isolates and percent similarity, with notes on reported DON degradation/biocontrol relevance for colony isolates obtained from DON transforming samples V15, V4, Z13, Z1T19 and C4 [25,30,31,32,33,34,52,64,65,66,67,68]; Table S4: Antibiotics and working concentrations applied to Z13 pre-inocula for community simplification.

## Abbreviations

The following abbreviations are used in this manuscript:

ASV	Amplicon Sequence Variant
3-ADON	3-acetyl-deoxynivalenol
15-ADON	15-acetyl-deoxynivalenol
DMEM	Dulbecco’s Modified Eagle Medium
DOM-1	De-epoxy-deoxynivalenol
DON	Deoxynivalenol
DON-3G	Deoxynivalenol-3-glucoside
ESI	Electrospray ionization
HPLC-UV	High-performance liquid chromatography with ultraviolet detection
LC-HRMS	Liquid chromatography–high-resolution mass spectrometry
MS/MS	Tandem mass spectrometry
NADPH	Nicotinamide adenine dinucleotide phosphate (reduced form)
PCR	Polymerase chain reaction
PQQ	Pyrroloquinoline quinone
QIIME 2	Quantitative Insights Into Microbial Ecology 2
RT	Retention Time
TOF	Time-of-flight
UHPLC	Ultra-high-performance liquid chromatography
