# Supplementary material for: Chemical, Biological, and Ecological Evidence for Aerobic Deoxynivalenol Detoxification in Agronomic Soil-Derived Bacterial Communities
Source: Toxins (Basel). 2026 Jun 22;18(6):273. doi: 10.3390/toxins18060273 (PMC13307919; doi:10.3390/toxins18060273)
Supplement: Supplementary file 1 [file toxins-18-00273-s001.zip › toxins-4348288-supplementary.pdf]

Supplementary Materials: Chemical, Biological, and Ecological Evidence for Aerobic Deoxynivalenol Detoxification in Agronomic Soil-Derived Bacterial Communities

Natalia Martínez-Reyes, Rosa E. Cardoza, Estela Melcón-Fernández, Rafael Balaña-Fouce, Lea Brückner, Rocío Montes-Ruiz, Benedikt Cramer, Hans-Ulrich Humpf, Pedro A. Casquero and Santiago Gutiérrez

Supplementary Table S1. Soil sample metadata used for DON biodegradation screening. The table lists each sample code, DON degradation percentage with respect to the batch's control, crop type (e.g., beans, hop, vine), origin/collection location, *Trichoderma* presence/identity and collection date; entries include field soils and *in vitro* soil cultures inoculated with *Trichoderma* and harzianum A. N/A indicates that *Trichoderma* presence was not analyzed.

Code	DON degradation	Crop	Origin	*Trichoderma* presence	Collection date
Z1T19	80%	corn	In vitro culture of soil with *Trichoderma* spores T19	*T. arundinaceum*	3/27/2023
Z1HA	67%	corn	In vitro culture of soil with harzianum A	N/A	3/27/2023
Z2T19	96%	corn	In vitro culture of soil with *Trichoderma* spores T19	*T. arundinaceum*	3/27/2023
Z2HA	76%	corn	In vitro culture of soil with harzianum A	N/A	3/27/2023
Z6T19	95%	corn	In vitro culture of soil with *Trichoderma* spores T19	*T. arundinaceum*	3/27/2023
Z6HA	82%	corn	In vitro culture of soil with harzianum A	N/A	3/27/2023
C4	68%	vine	Peñafiel (Valladolid)	N/A	2/17/2023
C5	26%	vine	Peñafiel (Valladolid)	N/A	2/17/223
C6	68%	vine	Gordoncillo	N/A	3/16/223
C7	97%	vine	Gordoncillo	N/A	3/16/2023
C8	96%	vine	Gordoncillo	N/A	3/16/2023
C9	95%	vine	Gordoncillo	N/A	3/20/2023
C10	67%	vine	Gordoncillo	N/A	3/20/2023
V1	33%	vegetables	Villacelama	N/A	3/28/2023
Z11	81%	corn	Santas Martas	N/A	3/29/2023
C11	33%	vine	Villamañán	N/A	4/08/2023
V2	13%	vegetables	Revellinos de Campos (Zamora)	N/A	4/08/2023
P1	63%	grass	Revellinos de Campos (Zamora)	N/A	4/08/2023
Z12	46%	corn	Villamañán	N/A	4/08/2023
T1	98%	wheat	Micereces de Tera (Zamora)	N/A	4/09/2023
V3	97%	vegetables	San Adrián de Juarros (Burgos)	N/A	4/09/2023
V4	98%	vegetables	San Adrián de Juarros (Burgos)	N/A	4/09/2023
V5	98%	fallow	Valverde	N/A	4/12/2023

V6	50%	fallow	Villares de Órbigo	N/A	4/12/2023
T2	70%	wheat	Valverde	N/A	4/12/2023
Z13	98%	corn	Valdepolo	N/A	4/12/2023
V7	39%	peas	Castrotrierra de Valmadrigal	N/A	4/12/2023
V8	67%	barbecho	Valverde	N/A	4/15/2023
V9	18%	barbecho	Valverde	N/A	4/15/2023
V10	79%	barbecho	Castrocalbón	N/A	4/15/2023
T3	19%	wheat	Castrocalbón	N/A	4/15/2023
T4	33%	wheat	Castrocalbón	N/A	4/15/2023
V11	9%	rye	Castrocalbón	N/A	4/15/2023
V12	23%	fallow	Castrobol (Valladolid)	N/A	3/30/2023
V13	12%	fallow	Castrobol (Valladolid)	N/A	3/30/2023
V14	96%	fallow	Candamo (Asturias)	N/A	4/08/2023
V15	79%	fallow	Cabreros del Río	N/A	4/14/2023
V16	91%	fallow	Cabreros del Río	N/A	4/14/2023
V17	55%	vetch	Valverde	N/A	4/23/2023
V18	95%	oats	Valverde	N/A	4/23/2023
V19	97%	chickpeas	Villamañán	N/A	4/23/2023
H2	26%	hop	Carrizo	N/A	4/23/2023
Z14	26%	corn	Milles de la Polvorosa (Zamora)	N/A	4/23/2023

Supplementary Table S2. DON-related metabolites targeted by LC–HRMS, listing compound name, molecular formula, and calculated monoisotopic exact mass.

Compound	Sum formula	Exact mass	Matches in Z13
Deoxynivalenol	$C_{15}H_{20}O_6$	296.1259	No
3-keto-deoxynivalenol	$C_{15}H_{18}O_6$	294.1103	No
3-epi-deoxynivalenol	$C_{15}H_{20}O_6$	296.1259	No
Deepoxy-deoxynivalenol	$C_{15}H_{20}O_5$	280.1311	formate adduct (m/z 326.1367) at RT 3.54 min
Acetyldeoxynivalenol	$C_{17}H_{20}O_7$	338.1366	RT 5.62 min
3,15-Diacetyldeoxynivalenol	$C_{19}H_{24}O_8$	380.1471	
Deoxynivalenol 3-glucuronide	$C_{21}H_{28}O_{12}$	472.1581	
Hydrolyzed epoxide DON*	$C_{15}H_{22}O_7$	314.1365	RT 0.87 and 2.84 min
9,10-Dihydrodeoxynivalenol	$C_{15}H_{22}O_6$	298.1416	RT 5.33 and 7.44 min
Deoxynivalenol sulfate	$C_{15}H_{20}O_9S$	374.0671	
Deoxynivalenol glucoside	$C_{21}H_{30}O_{11}$	458.1788	

*Hypothetical compounds proposed by BioTransformer 3.0 [63].

Supplementary Table S3. 16S rDNA BLAST/NCBI identification of colony isolates and percent similarity, with notes on reported DON degradation/biocontrol relevance for colony isolates obtained from DON transforming samples V15, V4, Z13, Z1T19 and C4.

Colony ID	Identification	Sim. %	DON degradation
V15-1	*Arthrobacter oryzae*	>99	Genus: biocontrol of mycotoxigenic fungi [64,65]. Found on consortia that can degrade DON [66].
V15-2	*Variovorax ureilyticus*	>99	Genus: biocontrol of mycotoxigenic fungi [67].
V15-3	*Variovorax boronicumulans*	>99	Previously described
V15-4	*Azospirillum brasilense*	>99	
V15-5	*Brevundimonas* sp.	>99	
V15-6	*Epilithonimonas lactis*	>99	
V15-7	*Variovorax boronicumulans*	>99	
V4-1	*Pseudomonas thivervalensis*	>99	Genus: DON degradation in consortia [25,33,34,52].
V4-2	*Ensifer adhaerens*	>99	
V4-3	*Pseudoxanthomonas mexicana*	--	Genus: DON degradation in consortia [34].
V4-4	*Agrobacterium tumefaciens*	>99	
V4-5	*Phyllobacterium ifriqiyense*	>99	
V4-6	*Pseudomonas atacamensis*	>99	Previously described
V4-7	*Pseudoxanthomonas mexicana*	>99	
V4-8	*Agrobacterium tumefaciens*	>99	
Z13-1	*Pseudomonas brassicacearum*	>99	Previously described
Z13-2	*Achromobacter spanius*	>99	DON degradation single strain [30] and in consortia [34,52,68].
Z13-3	*Ensifer adhaerens*	>99	
Z13-4	*Stenotrophomonas humi*	>99	Genus: DON degradation in consortia [31,32].
Z13-5	*Pseudomonas* sp.	>99	Previously described
Z13-6	*Flavobacterium johnsoniae*	>99	Genus: DON degradation in consortia [66].
Z13-7	*Microbacterium phyllosphaerae*	>99	
Z13-8	*Pseudomonas brassicacearum*	>99	Previously described
Z13-9	*Chryseobacterium* sp.	>99	
Z13-10	*Pseudomonas oryzihabitans*	>99	Previously described
Z13-11	*Chryseobacterium* sp.	>99	
Z13-12	*Shinella sedimenti*	>99	
Z13-13	*Sphingopyxis sp.*	>99	Genus: DON degradation in consortia [34,68].

Z13-14	*Pseudomonas brassicacearum*	>99	Previously described
Z1T19-1	*Methylorubrum extorquens*	>99	
Z1T19-2	*Ensifer adhaerens*	>99	
Z1T19-3	*Ensifer adhaerens*	>99	
Z1T19-4	*Pseudomonas oryzihabitans*	>99	Previously described
Z1T19-5	*Dermacoccus nishinomiyaensis*	>99	
Z1T19-6		>99	
Z1T19-7	*Achromobacter spanius*	>99	Previously described
Z1T19-8	*Sinorhizobium meliloti*	>99	
C4-1	*Chryseobacterium lathyri*	>99	
C4-2	*Stenotrophomonas rhizophila*	>99	Previously described
C4-3	*Stenotrophomonas rhizophila*	>99	Previously described
C4-4	*Paenarthrobacter nitroguajacolicus*	>99	
C4-5	*Achromobacter spanius*	>99	Previously described
C4-6	*Stenotrophomonas rhizophila*	>99	Previously described
C4-7	*Chryseobacterium lathyri*	>99	

Supplementary Table S4. Antibiotics and working concentrations applied to Z13 pre-inocula for community simplification.

**Antibiotic**	**Concentration (µg/mL)**
Ampicillin	50
Kanamycin	25
Chloramphenicol	25
Spectinomycin	50
Cefotaxime	100
Geneticin (G418)	50
Rifampicin	25
Tetracycline	25
Carbenicillin	50
Phleomycin	10
